# Body mass as a supertrait linked to abundance and behavioral dominance in hummingbirds: A phylogenetic approach

**DOI:** 10.1002/ece3.4785

**Published:** 2019-02-07

**Authors:** Rafael Bribiesca, Leonel Herrera‐Alsina, Eduardo Ruiz‐Sanchez, Luis A. Sánchez‐González, Jorge E. Schondube

**Affiliations:** ^1^ Posgrado en Ciencias Biológicas, Unidad de Posgrado, Coordinación del Posgrado en Ciencias Biológicas UNAM Mexico City Mexico; ^2^ Instituto de Investigaciones en Ecosistemas y Sustentabilidad Universidad Nacional Autónoma de México Morelia Mexico; ^3^ University of Groningen Groningen The Netherlands; ^4^ Departamento de Botánica y Zoología, Centro Universitario de Ciencias Biológicas y Agropecuarias Universidad de Guadalajara Zapopan México; ^5^ Museo de Zoología “Alfonso L. Herrera”, Depto. de Biología Evolutiva, Facultad de Ciencias Universidad Nacional Autónoma de México Ciudad de México México

**Keywords:** ancestral states, body mass, morphological traits, phylogenetic signal

## Abstract

Body mass has been considered one of the most critical organismal traits, and its role in many ecological processes has been widely studied. In hummingbirds, body mass has been linked to ecological features such as foraging performance, metabolic rates, and cost of flying, among others. We used an evolutionary approach to test whether body mass is a good predictor of two of the main ecological features of hummingbirds: their abundances and behavioral dominance. To determine whether a species was abundant and/or behaviorally dominant, we used information from the literature on 249 hummingbird species. For abundance, we classified a species as “plentiful” if it was described as the most abundant species in at least part of its geographic distribution, while we deemed a species to be “behaviorally dominant” when it was described as pugnacious (notably aggressive). We found that plentiful hummingbird species had intermediate body masses and were more phylogenetically related to each other than expected by chance. Conversely, behaviorally dominant species tended to have larger body masses and showed a random pattern of distribution in the phylogeny. Additionally, small‐bodied hummingbird species were not considered plentiful by our definition and did not exhibit behavioral dominance. These results suggest a link between body mass, abundance, and behavioral dominance in hummingbirds. Our findings indicate the existence of a body mass range associated with the capacity of hummingbird species to be plentiful, behaviorally dominant, or to show both traits. The mechanisms behind these relationships are still unclear; however, our results provide support for the hypothesis that body mass is a supertrait that explains abundance and behavioral dominance in hummingbirds.

## 1 INTRODUCTION

The uniqueness and complexity of ecological community dynamics can be described through comparative analysis of informative ecological traits (McGill, Enquist, Weiher, & Westoby, 2006; Webb, Hoeting, Ames, Pyne, & Poff, 2010). Body mass is considered a critical ecological and evolutionary trait given its capacity to explain a substantial amount of variability associated with different biological traits and ecological processes (Brown & Maurer, 1986; White, Ernest, Kerkhoff, & Enquist, 2007; Woodward, Ebenman, Emmerson, Montoya, & Olesen, 2005). Of these, body mass is related to animal energetics, lifespan duration, home range, and territory size (Woodward et al., 2005). Therefore, body mass matches the definition of a “supertrait”: an easy‐to‐measure trait that embraces the variation in biological, ecological, and evolutionary processes in a group of organisms (Madin et al., 2016).

In the case of birds, and specifically hummingbirds (Apodiformes, Trochilidae), body mass has proven to be a suitable predictor of a wide range of variables such as metabolic rate, flying costs, flower choice, abundance, and the outcome of aggressive interactions among individuals of the same or different species (del Arizmendi & Ornelas, 1990; McGill et al., 2006; Powers & Conley, 1994; Temeles, Koulouris, Sander, & Kress, 2009). Several studies have related hummingbird abundances and behavioral dominance to contrasting floral exploitation strategies that are associated with body mass (del Arizmendi & Ornelas, 1990; Lara, Lumbreras, & González, 2009; McGill et al., 2006). The existence of strong relationships among body mass, energetic needs, resource abundance, and resource partitioning allows us to relate the individual characteristics of species to the structure and dynamics of ecological communities (Blackburn et al., 1993; Lara et al., 2009; White et al., 2007; Woodward et al., 2005).

Body mass also has been proposed as a trait that helps explain behavioral dominance in hummingbirds, with larger bodied species tending to be more aggressive and dominant than smaller ones (Altshuler, 2006; McGill et al., 2006). Martin and Ghalambor (2014) analyzed body mass in the context of evolutionary distance among species to understand patterns of aggressive interspecific interactions in several bird groups, including hummingbirds. They found that species with larger body masses were dominant over smaller species during aggressive interactions. However, the advantages generated by a larger body size declined with an increase in evolutionary distance between interacting species, with smaller species that belong to different clades than the larger ones gaining some benefits from morphological or behavioral differences (Martin & Ghalambor, 2014). Hence, both body mass and phylogenetic distance could determine the outcome of aggressive interactions in hummingbirds, which in turn may influence the structure of their communities (Martin & Ghalambor, 2014). In that sense, incorporating phylogenetic information into ecological studies offers a framework for understanding the evolutionary history of a trait and the role that this trait could have played in establishing community assemblages (Emerson & Gillespie, 2008; Losos, 2008).

This evolutionary perspective relies on the idea that closely related species should be ecologically similar as they share morphological and ecological traits, either because they are inherited ancestral traits, or are independently evolved similar traits. On the other hand, ecological similarities may also arise through other mechanisms, such as phylogenetic niche conservatism, in which species maintain ancestral ecological characteristics at a given divergence point (Losos, 2008; Qian & Ricklefs, 2016; Wiens & Graham, 2005). In hummingbirds, morphological traits of body mass, closed wing length, and exposed culmen length, all show a phylogenetic signal, being similar among related species, suggesting that they are conserved traits (Blomberg, Garland, & Ives, 2003; Graham, Parra, Tinoco, Stiles, & McGuire, 2012; Losos, 2008). However, species that are not closely related can share traits due to convergent evolution, as for behavioral dominance (Arbuckle & Speed, 2016; Kraft, Cornwell, Webb, & Ackerly, 2007), but in this case a low phylogenetic signal would be expected. Although trait evolution and ecological pressures have been studied separately, the association of body mass with the capacity of a given species to become very abundant or behaviorally dominant remains a poorly explored topic.

In this study, we evaluated whether body mass can be used as a supertrait that explains the ecologically important traits of abundance and behavioral dominance in hummingbirds under an evolutionary framework. Given that body mass is closely related to abundance and tends to show a strong phylogenetic signal in several animal clades including hummingbirds (Graham et al., 2012; Kamilar & Cooper, 2013), this trait can be an important predictor of hummingbird abundance (Nee, Read, Greenwood, & Harvey, 1991; White et al., 2007). Hence, we expect that the most abundant species of hummingbirds will be similar in size and will show phylogenetic clustering, being more closely related species (Emerson & Gillespie, 2008, and Losos, 2008). On the other hand, we might expect that behavioral dominance (i.e., pugnacity) arose at different moments across the evolutionary history of this group as a widespread trait, and as a consequence, behaviorally dominant species will exhibit variation in body mass and present a pattern of phylogenetic evenness (i.e., aggressiveness as a convergent trait, Cavender‐Bares, Ackerly, Baum, & Bazzaz, 2004; Cavender‐Bares, Kozak, Fine, & Kembel, 2009; Kraft et al., 2007; Ingram & Shurin, 2009).

## 2 METHODS

### 2.1 Criteria for data collection

We collected information on hummingbird species to generate a database containing information on: geographic localities where each species has been studied, their abundances at these localities, behavioral dominance level (i.e., level of aggressiveness), morphological data of body mass and total body length, and presence of co‐occurring species. Data were gathered from published references in peer‐reviewed journals, specialist books, and available digital information (Clements et al., 2013; Johnsgard, 1997; Schuchmann, 1999; online: Cornell Lab of Ornithology and Lepage, 2014 in Avibase). All morphological data were collected from only one source (Schuchmann, 1999). Body mass is presented in grams (g), and body length in centimeters (cm). Body length data included in our database represent the length from the tip of the beak to the tip of the tail. To reduce between‐sex variation in morphological data, we only used data from male individuals following Ricklefs and Travis (1980).

We classified hummingbird species in terms of their abundance using a conservative approach, as there are no quantitative data on abundance (ind./ha) for most hummingbird species, and where data do exist it may be highly variable across geographic localities. Therefore, we determined abundance as a category and not as a continuous variable, classifying hummingbirds as being “plentiful” or “not plentiful.” Plentiful species were those species that clearly dominated their communities across part of their geographic distribution. This included species that were described as the "most abundant" or "very abundant" in one or more localities. As species vary in abundance among communities in response to several factors such as local environmental conditions, we assumed that plentiful species must be the most copious species in at least one locality with optimal environmental conditions. On the other hand, species classified as “not plentiful” were never the most abundant species of a locality, regardless of environmental conditions within their geographic distribution. Most of the species we classified as plentiful were the most abundant species in several localities, and when the number of localities where they were the most abundant hummingbird was low, there were several studies indicating their ecological dominance in these localities. For abundance data, we only included species that were part of communities that included three or more species. This approach, while allowing us to have a high level of confidence when we classified a species as plentiful, reduced our capacity to detect some species that are extremely abundant but for which there are no reliable data.

Furthermore, we considered species to be behaviorally dominant when they were described as aggressive ("most aggressive" or "notably aggressive”), with a substantial ability to chase other hummingbirds from foraging sites, and presented a marked ability to monopolize resources. As a result, our data set of behaviorally dominant species was composed of those species reported to be pugnacious in at least one study. Species reported solely as being “territorial” were not considered behaviorally dominant because territoriality does not always include all the elements considered in our definition of behavioral dominance, and hummingbirds are likely to defend territories mainly against members of their own species and not against all other species present in a community (Feinsinger & Colwell, 1978; Morse, 1974).

### 2.2 Molecular data and phylogenetic inference

We constructed a phylogenetic tree for 249 species of hummingbirds using one species in the family Apodidae as an outgroup (*Aerodramus salangana*; Apodiforme, Apodidae). To generate our phylogenetic hypothesis, we selected two nuclear genes [nDNA; beta‐fibrinogen intron 7 (Bifb) and adenylate kinase (AK1)] and two mitochondrial genes [mtDNA; NADH dehydrogenase subunit 2 and 5 (ND2, ND5)] (González‐Caro, Parra, Graham, McGuire, & Cadena, 2012). Sequences for the four genes were downloaded from GenBank (Supporting Information Appendix S1; Benson et al., 2013) and aligned using MUSCLE (Edgar, 2004). The best‐fit model of nucleotide substitution for each gene was calculated using the software jModeltest v. 2.1.6 (Darriba, Taboada, Doallo, & Posada, 2012). The four single matrices were concatenated using Mesquite software v. 3.03 (Maddison & Maddison, 2015). The phylogenetic relationships of the concatenate matrix were estimated under a Bayesian framework in MrBayes v. 3.2.6 (Ronquist & Huelsenbeck, 2003) on the CIPRES Science Gateway v. 3.3 (Miller, Pfeiffer, & Schwartz, 2010). We used one cold and three heated chains, set to run for 40,000,000 generations, with sampling every 2,000 generations. Stationarity was determined by the likelihood scores for time to convergence through the average standard deviation of splits frequencies, and 25% of the first sample points collected to stationarity were eliminated. A 50% majority‐rule consensus tree was obtained after burn‐in (following Graham, Parra, Rahbek, & McGuire, 2009; McGuire et al., 2014). Our phylogeny is in close agreement with that proposed by McGuire et al. (2014). It should be noted, however, that our phylogenetic tree is not intended as a new phylogenetic hypothesis of the group, instead it was built with the purpose of having a phylogeny including the largest possible number of hummingbird species to conduct analyses for this research.

### 2.3 Phylogenetic structure metrics

To determine whether hummingbird species that share a particular trait (e.g., plentifulness and behavioral dominance) are more closely related than expected by chance, we calculated two metrics: (a) the Net Relatedness Index (NRI) and (b) the Nearest Taxon Index (NTI). Both NRI and NTI are standardizations of the mean pairwise phylogenetic distance (MPD) and the mean nodal nearest distance (MNTD), respectively. Specifically, NRI is the average phylogenetic relatedness between all pairwise combinations of taxa, whereas NTI indicates the mean phylogenetic relatedness between each taxon and its nearest relative. NRI values in our analysis quantify the overall clustering of plentiful and behaviorally dominant species on the phylogeny, whereas NTI is more sensitive to the phylogenetic structure near the terminal nodes of the phylogeny, and measures the extent of terminal clustering, independently of the level of deep clustering in the tree (Li et al., 2014; Webb, Ackerly, & Kembel, 2008; Webb, Ackerly, McPeek, & Donoghue, 2002). NRI and NTI were first used in community ecology for comparing whether species in a given community can be regarded as a random set of species. Evidence for a nonrandom subset of species is then taken to make inferences on the nature of the assembly driver. Here, we applied these metrics to assess whether the set of dominant species is a random set of species within a phylogenetic tree. Therefore, by using NRI/NTI we can evaluate whether dominant species are scattered or clustered across a phylogeny. The calculations of these indexes were performed in the R software (version 3.2.1) scientific computing environment (R Development Core Team, 2015) with the package “picante” (Kembel et al., 2010).

We assessed the significance of these parameters through measures of the standardized effect size (SES effect), which describes the difference between phylogenetic distances in plentiful and dominant species in the observed phylogeny versus randomly generated null phylogenies (999 random draws). To perform this, we used the “shuffle distance matrix labels” null model method, which shuffles species labels across all taxa included in the phylogenetic tree (Li et al., 2014; Webb et al., 2008). Positive SES values of NRI or NTI indicate phylogenetic clustering with lower phylogenetic distances among survey species (Li et al., 2014). Negative SES values indicate phylogenetic evenness with greater phylogenetic distance among species than expected by chance (Li et al., 2014).

### 2.4 Phylogenetic signal

We assessed the existence of a phylogenetic signal in body mass and body length of hummingbirds by estimating Blomberg’s *K* and Pagel's λ statistics (Blomberg et al., 2003; Freckleton, Harvey, & Pagel, 2002; Losos, 2008; Pagel, 1999a; Revell, 2012), as quantitative measures of the tendency for related species to exhibit similar functional traits and ecological characteristics inherited from common ancestors (Blomberg et al., 2003; Kamilar & Cooper, 2013). Blomberg’s *K* is a measure of the degree to which phylogeny predicts ecological similarity of species. Where *K* > 1, species were considered to be more similar than expected under a Brownian motion model. A value of *K* = 1 indicated that there was a strong phylogenetic signal and the trait has evolved according to the Brownian motion model of evolution. Finally, if *K* = 0, there was considered to be no phylogenetic signal for the trait (Ackerly, 2009; Blomberg et al., 2003; Kamilar & Cooper, 2013; Paradis, Claude, & Strimmer, 2004).

Alternatively, Pagel's λ is a quantitative measure of phylogenetic dependence. This measure allowed us to determine whether the ecological similarity among species is associated with their phylogenetic relatedness and varies continuously from 0 to 1. A value of λ = 0 indicates that there is no phylogenetic signal in the trait (i.e., the trait has evolved independently of phylogeny, and thus, the trait of close relatives is no more similar on average than that of distant relatives), while a value of λ = 1 indicates that there is a strong phylogenetic signal under a Brownian motion model of evolution. Where intermediate values are obtained, the involved trait may have evolved according to processes other than Brownian motion (Kamilar & Cooper, 2013; Pagel, 1999b; Paradis, 2006; Revell, Harmon, & Collar, 2008). We estimated Blomberg's *K* and Pagel's λ using the R packages “picante” (Kembel et al., 2010) and “phytools” (Revell, 2012).

### 2.5 Phylogenetic generalized linear models

We used Phylogenetic Generalized Linear Models (PGLM) to perform phylogenetic logistic regressions. The method is based on an evolutionary model and incorporates binary variables of traits with values that switch between 0 and 1. To assess relationships among the two continuous variables of body mass and length, and the two binary variables of abundance (plentiful/not plentiful) and behavioral dominance (dominant/not dominant), we ran PGLMs with 999 bootstrap iterations using the package “phyloglm” (Ives & Garland, 2010; Revell, 2012) run in R (version 3.2.1; R Development Core Team, 2015).

### 2.6 Ancestral character state estimation

Ancestral reconstruction can be used to recover the values of the ancestral character states of a trait (Finarelli & Flynn, 2006; Joy, Liang, McCloskey, Nguyen, & Poon, 2016). In this study, we inferred the ancestral character state for body mass and body length using our phylogenetic tree for Trochilidae. We used a maximum‐likelihood (ML) estimation of ancestral states for categorical characters (presence/absence of plentifulness and behavioral dominance), and continuous characters (body mass, body length; Schluter, Price, Mooers, & Ludwig, 1997; Pagel, 1999b; McGuire, Witt, Altshuler, & Remsen, 2007; Revell, 2014). ML indicates the probability of alternative character states using branch lengths to model the trait change rate along each branch. To calculate the ML of categorical data, we implemented the Markov k‐state 1 parameter model, in which both “forward” and “backward” transitions rates are considered as equal. We used Mesquite to conduct these analyses (Maddison & Maddison, 2015). Continuous traits were analyzed using “phytools” (Revell, 2012) for R 3.2.1 (R Development Core Team, 2015).

### 2.7 Body mass and evolutionary distinctiveness

The evolutionary distinctiveness (ED) index measures the contribution made by different species to the phylogenetic diversity of a group (Isaac, Turvey, Collen, Waterman, & Baillie, 2007). Scores of this metric reflect differences among species and can be used to presume taxonomic changes. Moreover, this index is independent of the size of the clades in the phylogeny, allowing the comparison of organisms grouped at different taxonomic levels, and it is also sensitive at the phylogeny tips, allowing the inference of evolutionary changes in species characters (Cavin & Kemp, 2011; Isaac et al., 2007; Redding, DeWolff, & Mooers, 2010). We used this metric to determine how phylogenetic diversity is related to body mass in hummingbirds, and the role that both ED and body mass may have on being plentiful or behaviorally dominant. To obtain ED scores, we used the package “picante” (Kembel et al., 2010) for the R software. The correlation analysis between ED and body mass was performed using GraphPad Prism v.7.

## 3 RESULTS

### 3.1 Plentiful and behaviorally dominant species

**Figure 1:**
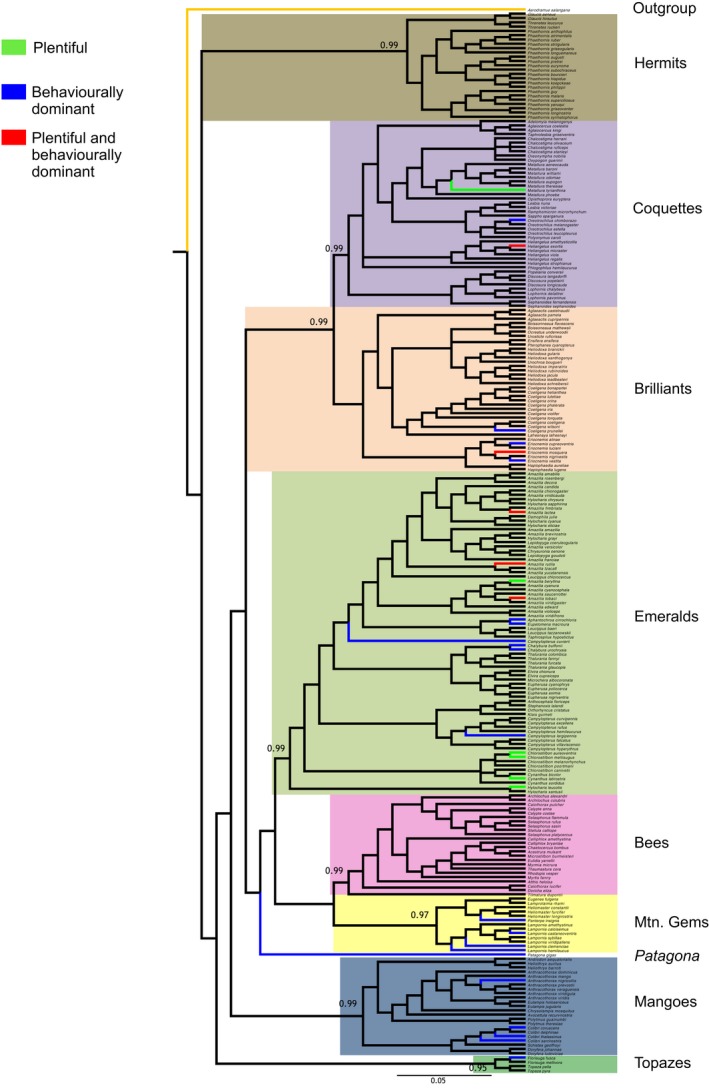
Bayesian 50% majority consensus tree based on the concatenated nuclear and mitochondrial genes (Ak1, Bfib, ND2, and ND5), showing the distribution of abundant and dominant species. Green branches represent plentiful species, blue branches indicate behaviorally dominant species, while red branches indicate species that are both plentiful and behaviorally dominant. Black branches represent nonplentiful, nonbehaviorally dominant species

From the 249 species included in our analyses, we found that 33 species of hummingbirds were plentiful or behaviorally dominant. These species were distributed in 20 genera within eight clades and the two subfamilies of Trochilidae: Phaethornithinae (Hermits) and Trochilinae (Trochilids; Figure 1). Eleven species complied with the prerequisites to be considered as plentiful, and 27 as behaviorally dominant, while five species presented both traits: Sapphire‐spangled Emerald (*Amazilia lactea*), Cinnamon Hummingbird (*A. rutila*), Copper‐rumped Hummingbird (*A. tobaci*), Golden‐breasted Puffleg (*Eriocnemis mosquera*), and Tourmaline Sunangel (*Heliangelus exortis*). The genera with the highest number of plentiful or behaviorally dominant species were *Amazilia* (*n* = 4), *Eriocnemis* (*n* = 3), *Colibri* (*n* = 3), and *Lampornis* (*n* = 3). As a clade, the Emeralds had the highest number of plentiful or behaviorally dominant species, containing 42.4% of all species that displayed one, or both, of the two traits (Table 1). The Bees clade was the only group that did not include plentiful or behaviorally dominant species.

**Table 1:** Plentiful (P) and behaviorally dominant (BD) species and their clades (McGuire et al., 2014). Values presented are means ± *SD*. Body length and body mass data are from male individuals. All data collected from Schuchmann (1999)

Clade	Species	BD	P	Body length (cm)	Body mass (g)
Emeralds	*Amazilia beryllina*		*	9.0 ± 1.0	4.4 ± 0.8
	*Amazilia lactea*	*	*	9.5 ± 1.2	4.0 ± 0.6
	*Amazilia rutila*	*	*	10.0 ± 2.0	5.0 ± 0.9
	*Amazilia tobaci*	*	*	10.0 ± 1.8	4.6 ± 0.8
	*Cynanthus latirostris*		*	9.5 ± 1.5	3.5 ± 0.6
	*Hylocharis leucotis*		*	9.5 ± 0.9	3.6 ± 0.7
	*Chlorostilbon mellisugus*		*	7.5 ± 1.0	3.5 ± 0.5
	*Chlorostilbon aureoventris*		*	10 ± 1.5	4.0 ± 0.9
	*Chalybura buffonii*	*		11.25 ± 1.5	7.1 ± 0.4
	*Chalybura urochrysia*	*		11.25 ± 1.5	7.1 ± 0.5
	*Campylopterus largipennis*	*		13.95 ± 1.9	9.5 ± 1.0
	*Campylopterus cuvierii*	*		12.25 ± 1.5	10.1 ± 0.6
	*Aphantochroa cirrochloris*	*		12 ± 1.2	9 ± 0.7
	*Eupetomena macroura*	*		16 ± 2.0	8.5 ± 1.0
Brilliants	*Eriocnemis vestitus*	*		9.5 ± 1.0	4.8 ± 0.8
	*Eriocnemis cupreoventris*	*		9.5 ± 1.0	5.6 ± 0.3
	*Eriocnemis mosquera*	*	*	12.5 ± 1.0	5.5 ± 0.3
	*Coeligena prunellei*	*		13.5 ± 0.8	6.35 ± 0.7
Mangoes	*Colibri coruscans*	*		13.5 ± 1.0	8.1 ± 0.8
	*Colibri serrirostris*	*		12.5 ± 1.0	6.2 ± 0.12
	*Colibri thalassinus*	*		11 ± 1.0	5.7 ± 0.7
	*Anthracothorax nigricollis*	*		11.5 ± 1.0	6.9 ± 0.8
Mtn. Gems	*Panterpe insignis*	*		10.75 ± 0.5	6.05 ± 0.3
	*Lampornis castaneoventris*	*		10.75 ± 1.5	5.95 ± 0.5
	*Lampornis clemenciae*	*		13 ± 1.1	8 ± 0.4
	*Lampornis hemileucus*	*		10.5 ± 1.0	6.2 ± 0.7
Coquettes	*Leucochloris albicollis* a	*		10.75 ± 1.5	5 ± 0.5
	*Heliangelus exortis*	*	*	10.5 ± 1.0	4.5 ± 0.2
	*Oreotrochilus chimborazo*	*		13 ± 1.1	7.95 ± 0.3
	*Metallura tyrianthina*		*	9.5 ± 1.5	3.3 ± 0.5
Topazes	*Florisuga fusca*	*		12.5 ± 1.0	9 ± 0.6
	*Patagona* *Patagona gigas*	*		21 ± 2.0	19.3 ± 1.7
Hermits	*Ramphodon naevius* a	*		15 ± 1.0	6.9 ± 2.9

* Indicates the presence of the trait in the species.

a No molecular data.

### 3.2 Molecular data and phylogenetic inference

The models obtained for each gene matrix were: TPM2uf+I + G (Bfib); F81+I + G (AK1) and TPM3uf+I + G (ND2); F81+I + G (ND5). The BI tree recovered, with high support, all of the main clades reported by McGuire et al. (2007) (Hermits, Topazes, Mangoes, Coquettes, Mountain Gems, Emeralds, Brilliants, Bees and *Patagona*; Figure 1).

### 3.3 Phylogenetic analyses

Plentiful species exhibited phylogenetic clustering with positive values for both NRI and NTI, suggesting that they are more related phylogenetically than expected by chance (NRI = *p* < 0.003; NTI = *p* < 0.004). Hummingbird species with this trait span three hummingbird clades (Emeralds, Brilliants and Coquettes). On the other hand, behaviorally dominant species were randomly distributed along the phylogeny, having negative values of NRI with greater phylogenetic distance among species than expected by chance (*p* = 0.09).

### 3.4 Phylogenetic signal

Both Blomberg's *K* and Pagel's λ statistics showed a strong phylogenetic signal in body mass (*K* = 1.048, *p* = 0.01 and λ = 0.999, *p* = 0.001). We found low phylogenetic signal on body length in Blomberg's statistic (*K* = 0.462, *p* = 0.01) and a strong phylogenetic signal in Pagel's statistic (λ = 0.985, *p* = 0.001).

### 3.5 Phylogenetic generalized linear models

We found a statistically significant relationship between behavioral dominance and body mass (PGLM, estimate = 0.226, *SE* = 0.082, *z* value = 0.082, *p* = 0.006). We did not find a significant relationship when we conducted the same analysis for plentiful species.

### 3.6 Ancestral state estimation

**Figure 2:**
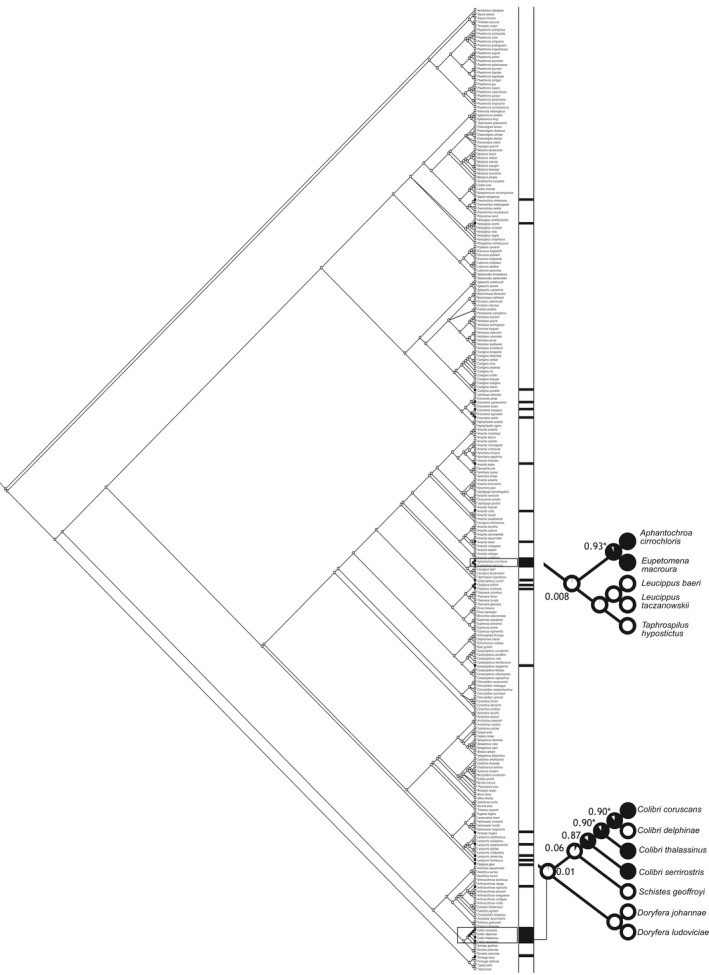
Maximum‐likelihood Markov model (Mkl) of ancestral state reconstructions describing behavioral dominance in hummingbirds. Pies show behavioral dominance, varying from all white (no dominance) to all black (highest dominance). Asterisk on pie diagrams indicates a likelihood higher than 0.89 (MK1‐ML) on phylogenetic reconstructions. Black horizontal bars correspond to observed data of behavioral dominance

The ancestral state analysis revealed that under a ML Markov model (Mk1 = 0.90), behavioral dominance is the ancestral condition in two hummingbird clades (Emeralds: genera *Aphantochroa* and *Eupetomena*) and Mangoes (genus *Colibri*; Figure 2). A total of four species within both clades exhibited a statistically significant value associated with a behaviorally dominant ancestry according to the Mk1 optimization model (see nodes marked with an asterisk on Figure 2). We did not find a pattern in the ancestral state reconstruction for the plentiful trait.

**Figure 3:**
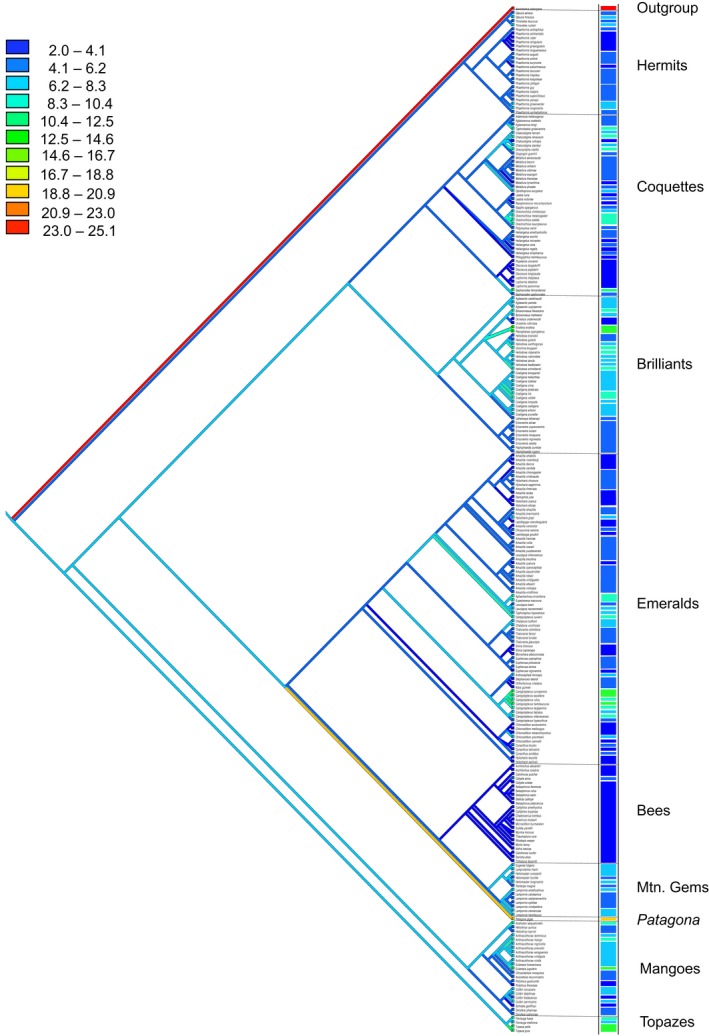
Ancestral state reconstruction by maximum likelihood (ML) of body mass in hummingbirds represented with a color gradient ranging from low (colder tones) to high (warmer tones) body mass

**Figure 4:**
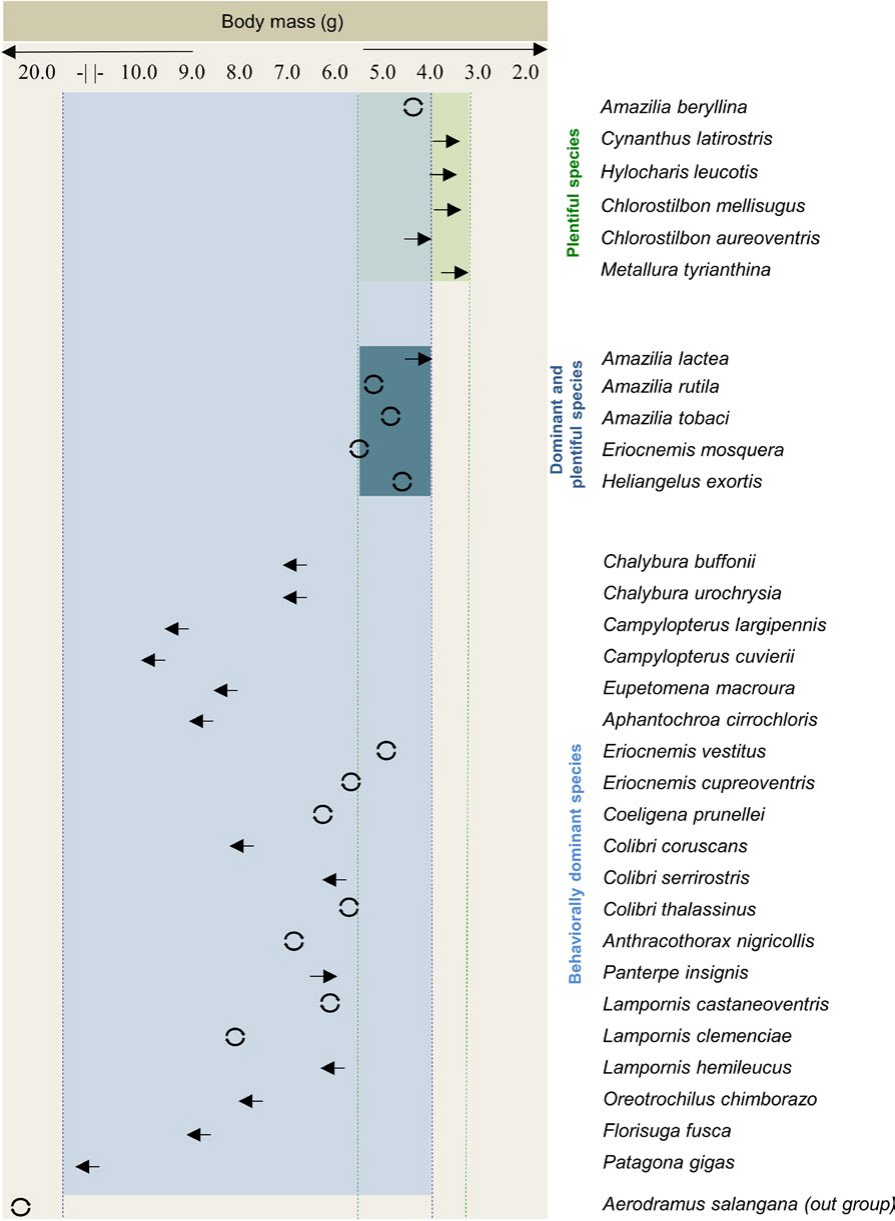
Average body mass of plentiful, behaviorally dominant, and both behaviorally dominant and plentiful species of hummingbirds. Arrows pointing to the left indicate greater body mass relative to the ancestral state, circles indicate a conserved body mass with regard to the ancestor, while arrows pointing to the right represent a decrease in body mass in relation to the ancestral state.

The ML reconstructions of body mass ancestral state indicated that 62.5% of the plentiful species possessed lower body masses than their ancestors. Furthermore, 46.1% of the species with behavioral dominance showed an increased in body mass in relation to their ancestors. Overall for our species pool, the ancestral reconstruction showed a reduction in body mass from 8.3 to 6.2 g. However, the Bee clade showed a larger reduction in body mass from 8.3 to 4.1 g. Conversely, *Patagona gigas* had an increase in body mass in relation to its ancestral state from 8.3 to 19 g (Figures 3 and 4). We did not find any pattern in body length.

### 3.7 Evolutionary distinctiveness

**Figure 5:**
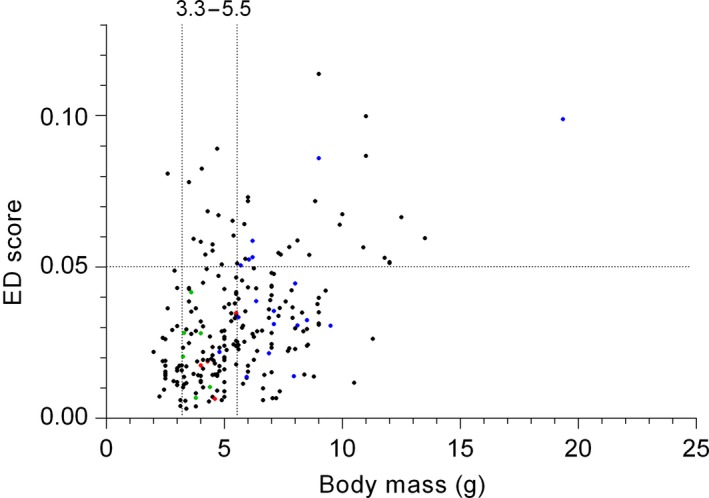
Relation between evolutionary distinctiveness (ED) scores and body mass in hummingbirds. ED scores showed a positive relationship with body mass, however, most species presented low ED scores. Green dots represent plentiful species, blue dots represent behaviorally dominant species, and red dots represent species that are both plentiful and behaviorally dominant. Plentiful species and species that are both plentiful and behaviorally dominant are found within the same range of body mass and show ED scores below 0.05. Vertical dotted lines represent the range of body mass values associated to plentiful species and species that are both plentiful and behaviourally dominant

The species with the highest ED score was the Tooth‐billed Hummingbird (*Androdon aequatorialis*) with an ED score of 0.113, which was even higher than that for the Giant Hummingbird (*Patagona gigas*; ED = 0.098). Overall, our 33 focal hummingbird species had an average ED score of 0.025. The ED scores for all species (*N* = 249) were positively correlated to their body mass (Spearman *r* = 0.36, *N* = 249, *p* = 0.0001). This pattern held even when we removed the Giant Hummingbird (*Patagona gigas*; Spearman *r* = 0.46, *N* = 30, *p* = 0.009; Figure 5). While ED scores showed a positive relationship with body mass, species showing ED scores below 0.05 exhibited a large diversity of body masses (2.0–11.5 g). Species that are plentiful (with a body mass ranging from 3.3 to 5.5 g), or that are both plentiful and behaviorally dominant (ranging in body mass from 4.0 to 5.5 g) exhibited low ED scores (from 0.006 to 0.04), while behaviorally dominant species (body mass from 4.0 to 19.3 g) exhibited a wide range of ED scores (0.015–0.10). This suggests that body mass is more important than ED to determine if a species is plentiful, behaviorally dominant, or if the species show both traits.

## 4 DISCUSSION

Our study found that hummingbird species classified as plentiful had intermediate body mass that was lower than the ancestral state and were phylogenetically clustered. By comparison, behaviorally dominant species had large body mass, having increased from the ancestral trait, and occurred randomly in the phylogeny indicating stochasticity or convergent evolution (Cavender‐Bares et al., 2004). Body mass also had a strong phylogenetic signal. This indicates that body mass is a supertrait that provides information on the abundance and behavioral dominance of hummingbirds when analyzed using a phylogenetic perspective. In this section, we first discuss the relationship between body mass and abundance. Second, we focus on the association between body mass and behavioral dominance. Finally, we consider why some species present both traits.

### 4.1 Plentiful species and body size

Our evolutionary approach provides insightful information to understand why some hummingbird species are plentiful while other species are not. Plentiful species in our study are more closely related phylogenetically than expected by chance and are mostly located within the Emeralds’ clade, with a small number of plentiful species being found in the clades of the Coquettes and Brilliants. This supports our predictions that plentiful species should exhibit similar body mass and be clustered in the phylogeny. However, our definition of plentiful included only 11 species, and our results should be considered as a hypothesis to be tested when abundance data are available for most hummingbird species.

Studies by Nee et al. (1991) and Blackburn et al. (1993) of different bird groups have found a positive relationship between abundance and body mass at low taxonomic levels (e.g., tribe, genus), and a negative relationship at higher taxonomic levels (e.g., order, class). Nee et al. (1991) speculated that at lower taxonomic levels, larger species might present an advantage in interspecific competition, which may cause a reduction in abundance in smaller‐sized species. This pattern tends to be stronger when the taxonomic group encompasses an entire guild, as in the case of hummingbirds (Nee et al., 1991). Our results for hummingbirds at the family level are similar to these studies in that hummingbird species that are plentiful were of intermediate size. This could reflect resource competition among species different body mass, and the physiological constraints faced by small‐sized hummingbirds (Powers & Conley, 1994; Suarez & Gass, 2002). However, in our study, large hummingbird species were not more plentiful. Therefore, two nonmutually exclusive hypotheses could explain this: (a) our study uses a broad measure of abundance that is closer to the concept of "ecological density" (sensu Blackburn & Gaston, 1997) than to crude density values used in other studies (Blackburn et al., 1993; Blackburn & Gaston, 1997; Nee et al., 1991). Moreover, (b) we used a higher taxonomic level in our study (family). This could generate enough differences in body mass among taxa to reduce the advantages that a large body size offers when trying to obtain and control food resources (Martin & Ghalambor, 2014; Nee et al., 1991).

Our results are comparable to those from studies that have found a polygonal relationship between abundance and body size, where abundance peaks at intermediate body mass for different groups of organisms at both regional and local spatial scales (Blackburn et al., 1993; Blackburn & Gaston, 1997; White et al., 2007). These studies suggest that under certain environmental conditions, intermediate body mass could provide some ecological advantages that allow organisms of intermediate size to be plentiful. This could be the case for hummingbirds and agrees with our finding of a reduction in body mass from the ancestral state for plentiful species. Hummingbirds with intermediate body size could benefit from being able to use flowers with a broad range of corolla sizes, while small and large‐sized hummingbird species tend to be restricted to use small or large‐sized flowers (Maglianesi, Blüthgen, Böhning‐Gaese, & Schleuning, 2014, 2015; Temeles et al., 2009). This advantage could be reflected ecologically by presenting higher abundances for intermediate sized species that do not share the behavioral and physiological limitations of smaller species (Maglianesi, Blüthgen, Böhning‐Gaese, & Schleuning, 2014, 2015). Additionally, while small‐sized species face higher energetic costs due to thermoregulation and hovering (Powers & Conley, 1994; Suarez & Gass, 2002), and large‐sized species present higher total energy needs and higher flying costs (Temeles et al., 2009), intermediate sized species could be energetically less constrained, and thus more abundant (Blackburn & Gaston, 1997). Hence, our results highlight the importance of lower and upper limits in body mass as a driver of abundance.

We also found that abundant species are phylogenetically clustered, and mostly located within the Emerald clade, with a lower number of species found in the Coquette and Brilliant clades. These results suggest that through their evolutionary history, taxa in the Emerald clade may have been exposed to different selective pressures, acquiring functional traits that allowed some species to be the most abundant hummingbirds in specific environments. For instance, several studies conducted at both local and regional scales indicate that Emerald species interact with a more significant number of plant species than hummingbirds belonging to other clades found in the same geographic areas (Feinsinger & Swarm, 1982; Lara‐Rodríguez et al., 2012; Ortiz‐Pulido, Díaz, Valle‐Díaz, & Fisher, 2012; Partida‐Lara et al., 2018). Additionally, Emeralds have been described as habitat generalists, capable of colonizing a broad range of habitats using both humid and xeric environments at different elevations, from the southern part of North America to Argentina (Bleiweiss, 1998; Graham et al., 2009; Ornelas, González, & Espinosa de los Monteros A, Rodríguez‐Gómez F, García‐Feria LM, 2014; Schuchmann, 1999). Graham et al. (2009) suggested that the ability of Emeralds to use a wide diversity of habitats, some of them restricted to other hummingbird clades, may be the result of physiological adaptations such as a higher capacity to maintain water balance that allowed them to reduce dehydration when nectar is not abundant and/or the environment is dry (Bakken, McWhorter, Tsahar, & Martinez del Rio, 2004). These physiological abilities could help explain why most of the abundant species are located within this clade (Lara‐Rodríguez et al., 2012).

### 4.2 Behavioral dominance and body mass

Except for the Bees, the remaining eight of the nine principal hummingbird clades (Hermits, Topazes, Mangoes, Brilliants, Coquettes, *Patagona*, Mountain Gems, and Emeralds) include at least one behaviorally dominant species. The Bee clade alone lacked either plentiful or behaviorally dominant species. This result could be a reflection of the species selection criteria applied in our study as we had to exclude some species of this clade from our analyses since in most cases they were members of only one‐ or two‐species communities. However, Bee hummingbirds represent the only clade present at the northernmost limit of the global hummingbird geographic distribution, with species living in the USA and Canada conducting latitudinal migratory movements (Carpenter, Hixon, Russell, Paton, & Temeles, 1993; Kodric‐Brown & Brown, 1978; López‐Segoviano, Bribiesca, & Arizmendi, 2018). Furthermore, members of this clade are not acting as plentiful or behaviorally dominant species in more complex communities located in Mexico and Central America, where they are winter migrants (Lara et al., 2009; López‐Segoviano et al., 2018; Schondube, 2012). This could be caused by the fact that a trade‐off appears to limit the ability of species to be good at both migration and their ability to win in aggressive interactions, allowing resident species to be dominant over migrant species (DesGranges & Grant, 1980; Freshwater, Ghalambor, & Martin, 2014; Martin & Ghalambor, 2014). However, the role of body mass in determining the structure of hummingbird communities composed only by members of the Bee clade requires further study.

Our prediction that behaviorally dominant species should show a random distribution in the phylogeny was supported by the evolutionary metrics (Mk1 and ED) calculated for our data. Overall, behaviorally dominant species showed large and intermediate body masses in the Emeralds and Coquettes, and intermediate values of this trait in the Brilliants and the Mangos. Our findings support the work of Martin and Ghalambor (2014) and Márquez‐Luna, Lara, Corcuera, and Valverde (2018) who propose that in order to win aggressive interactions independently of differences in body mass, species should be genetically distant so that the “disadvantage of being small in aggressive interactions could be overcome over evolutionary time through the accumulation of novel traits that can counteract the advantages of being large” (Martin & Ghalambor, 2014). The fact that behaviorally dominant species are present in small numbers in most clades further suggests a convergent process of behavioral evolution in hummingbirds (Kamilar & Cooper, 2013; Kraft et al., 2007; Losos, 2008).

We also found that all aggressive species had a body mass larger than 4.0 g. While this result could be an artifact of the lack of behavioral dominance in the Bee clade (see above), our results suggest that this is not the case. When body mass distribution is analyzed by clade, behaviorally dominant species are not associated with the large‐sized species in all clades. Additionally, while several species of the Emeralds, Coquettes, and Hermits exhibit a body mass smaller than 4.0 g, none of these showed behavioral dominance. This indicates that there is a lower limit on body mass associated with behavioral dominance in hummingbirds. This body mass threshold could be associated with a minimum muscle mass and strength required to chase and fight against other hummingbirds (Martin & Ghalambor, 2014; Morse, 1974; Peters, 1983). While several studies have suggested that behavioral dominance is related to large body sizes in hummingbirds (Ewald, 1985; Lara et al., 2009), our results show a considerable variation in body mass across behaviorally dominant species when the whole family is taken into account, and not only the members of a single community.

### 4.3 Species that are plentiful and exhibit behavioral dominance

Our results show a link between body mass range and the presence of both abundance and behavioral dominance traits in some hummingbird species. While plentiful species had a lower intermediate body mass and behaviorally dominant species had larger body mass, hummingbird species that presented both traits had a body mass between 4.0 and 5.5 g, falling within the overlap range of the two body mass distributions for each ecological trait. This suggests the existence of physiological/morphological trade‐offs associated with body size that limit being both plentiful and dominant in aggressive interactions. Nevertheless, the mechanisms behind this relationship are still unclear.

The association of body mass with the ecological traits of abundance and behavioral dominance found in our phylogenetic analysis provides a basis to understand the role of different hummingbird species within their ecological communities. Our findings suggest that the most abundant species in hummingbird communities could be expected to have intermediate body mass (3.3–5.5 g), while behaviorally dominant species should have a body mass above 4.0 g. While the body mass data in our study are limited to only males, we sampled 249 species of hummingbirds (≅74% of all extant species), thereby enabling an educated assessment of the ecological role of a species based on body mass. The patterns determined in our study provide a novel framework for generating hypotheses that associate body mass with physiological/morphological advantages, and promote discussion of the use of trait‐based and supertrait approaches to understand the mechanisms that determine the structure and composition of hummingbird communities.

## CONFLICT OF INTEREST

None declared.

## AUTHORS’ CONTRIBUTION

R.B and J.E.S conceived the idea and designed the methodology. Data collection was conducted by R.B. Data analyses were discussed and performed by R.B, E.R‐S, L.H‐A, and L.A.S‐G. While all authors contributed with ideas, data interpretation, and the process of writing of the manuscript, R.B and J.E.S led these two parts of the work. All authors contributed critically to the final draft and gave final approval for publication.

## Data Availability

Data available from the Dryad Digital Repository: https://doi.org/10.5061/dryad.2v4m067
