# Supplementary material for: Body mass as a supertrait linked to abundance and behavioral dominance in hummingbirds: A phylogenetic approach
Source: Ecol Evol. 2019 Feb 7;9(4):1623–37. doi: 10.1002/ece3.4785 (PMC6392494; doi:10.1002/ece3.4785)
Supplement: Supplementary file 1 [file ECE3-9-1623-s001.docx]

**Appendix**. Species names and GenBank accession numbers used in this study.

| *Hermits* |  |  |  |  |
| --- | --- | --- | --- | --- |
| Specie | *ND2* | *ND5* | *AK1* | *Bfib* |

| *Glaucis aeneus* | EU042554.1 | − | EU042470.1 | EU042390.1 |
| --- | --- | --- | --- | --- |
| *Glaucis hirsutus* | AY830486.1 | − | AY830561.1 | AY830637.1 |
| *Threnetes ruckeri* | AY830527.1 | − | AY830602.1 | AY830678.1 |
| *Threnetes leucurus* | AY830526.1 | − | AY830601.1 | AY830677.1 |
| *Phaethornis hispidus* | AY830512.1 | − | AY830587.1 | AY830663.1 |
| *Phaethornis yaruqui* | EU042584.1 | − | EU042499.1 | EU042419.1 |
| *Phaethornis guy* | AY830511.1 | − | JQ267540.1 | AY830662.1 |
| *Phaethornis syrmatophorus* | EU042583.1 | − | EU042498.1 | EU042418.1 |
| *Phaethornis koepckeae* | AY830513.1 | − | AY830588.1 | AY830664.1 |
| *Phaethornis philippii* | EU042581.1 | − | EU042496.1 | EU042416.1 |
| *Phaethornis bourcieri* | EU042577.1 | − | EU042492.1 | EU042412.1 |
| *Phaethornis longirostris* | KF525868.1 | − | EU042494.1 | EU042417.1 |
| *Phaethornis superciliosus* | KF525874.1 | − | − | − |
| *Phaethornis malaris* | JQ445680.1 | − | − | GU167143.1 |
| *Phaethornis anthophilus* | AY830514.1 | − | AY830589.1 | AY830665.1 |
| *Phaethornis longuemareus* | EU042574.1 | − | EU042489.1 | EU042409.1 |
| *Phaethornis griseoventer* | EU042580.1 | − | EU042495.1 | EU042415.1 |
| *Phaethornis atrimentalis* | EU042575.1 | − | EU042490.1 | EU042410.1 |
| *Phaethornis striigularis* | FJ231596.1 | − | − | − |
| *Phaethornis griseogularis* | EU042578.1 | − | EU042493.1 | EU042413.1 |
| *Phaethornis ruber* | AY830515.1 | − | − | AY830666.1 |
| *Phaethornis subochraceus* | GU167248.1 | − | GU167193.1 | GU167137.1 |
| *Phaethornis augusti* | EU042576.1 | − | EU042491.1 | EU042411.1 |
| *Phaethornis pretrei* | GU167247.1 | − | GU167192.1 | GU167136.1 |
| *Phaethornis eurynome* | GU167245.1 | − | GU167191.1 | GU167135.1 |
| *Eutoxeres aquila* | AY830483.1 | − | AY830558.1 | AY830634.1 |
| *Eutoxeres condamini* | AY830484.1 | − | AY830559.1 | AY830635.1 |

| *Topazes* |  |  |  |  |
| --- | --- | --- | --- | --- |
| Specie | *ND2* | *ND5* | *AK1* | *Bfib* |

| *Florisuga mellivora* | AY830485.1 | KC858536.1 | AY830560.1 | AY830636.1 |
| --- | --- | --- | --- | --- |
| *Florisuga fusca* | GU167229.1 | − | GU167175.1 | GU167119.1 |
| *Topaza pella* | JQ445903.1 | − | GU167198.1 | AY830679.1 |
| *Topaza pyra* | JQ445905.1 | − | − | − |

| *Mangoes* |  |  |  |  |
| --- | --- | --- | --- | --- |
| Specie | *ND2* | *ND5* | *AK1* | *Bfib* |

| *Androdon aequatorialis* | AY830463.1 | − | AY830539.1 | AY830614.1 |
| --- | --- | --- | --- | --- |
| *Doryfera ludovicae* | AY830477.1 | − | JQ267537.1 | AY830628.1 |
| *Doryfera johannae* | KC858416.1 | − | EU042465.1 | EU042383.1 |
| *Colibri delphinae* | EU042543.1 | − | EU042461.1 | EU042379.1 |
| *Colibri thalassinus* | EU042544.1 | KC858540.1 | EU042462.1 | EU042380.1 |
| *Colibri coruscans* | AY830476.1 | − | GU166829.1 | AY830627.1 |
| *Colibri serrirostris* | GU167222.1 | − | GU167168.1 | GU167112.1 |
| *Anthracothorax viridigula* | EU983413.1 | − | − | − |
| *Anthracothorax prevostii* | EU983409.1 | KC858541.1 | GU167155.1 | GU167099.1 |
| *Anthracothorax nigricollis* | EU983424.1 | − | EU042445.1 | EU042363.1 |
| *Anthracothorax veraguensis* | EU983402.1 | − | − | − |
| *Anthracothorax dominicus* | EU983417.1 | − | − | − |
| *Anthracothorax viridis* | EU983419.1 | − | − | − |
| *Anthracothorax mango* | AY830464.1 | − | AY830540.1 | AY830615.1 |
| *Avocettula recurvirostris* | EU983394.1 | − | GU167156.1 | GU167100.1 |
| *Eulampis jugularis* | EU983447.1 | − | AY830557.1 | AY830633.1 |
| *Eulampis holosericeus* | AY830530.1 | − | − | AY830681.1 |
| *Chrysolampis mosquitus* | EU042540.1 | − | EU042458.1 | EU042376.1 |
| *Polytmus guainumbi* | EU042585.1 | − | EU042500.1 | EU042420.1 |
| *Polytmus theresiae* | EU042586.1 | − | EU042501.1 | EU042421.1 |
| *Schistes geoffroyi* | AY830521.1 | − | AY830536.1 | AY830672.1 |
| *Heliothryx barroti* | AY830494.1 | − | AY830569.1 | AY830645.1 |
| *Heliothryx auritus* | GU167235.1 | − | GU167181.1 | GU167125.1 |

| *Brilliants* |  |  |  |  |
| --- | --- | --- | --- | --- |
| Specie | *ND2* | *ND5* | *AK1* | *Bfib* |

| *Heliodoxa xanthogonys* | EU042560.1 | − | EU042476.1 | EU042396.1 |
| --- | --- | --- | --- | --- |
| *Heliodoxa gularis* | GU167233.1 | − | GU167179.1 | GU167123.1 |
| *Heliodoxa branickii* | AY830490.1 | − | AY830565.1 | AY830641.1 |
| *Heliodoxa schreibersii* | EU042559.1 | − | EU042475.1 | EU042395.1 |
| *Heliodoxa rubinoides* | EU042558.1 | − | EU042474.1 | EU042394.1 |
| *Heliodoxa jacula* | AY830491.1 | − | AY830566.1 | AY830642.1 |
| *Heliodoxa imperatrix* | EU042557.1 | − | EU042473.1 | EU042393.1 |
| *Heliodoxa leadbeateri* | AY830492.1 | − | AY830567.1 | AY830643.1 |
| *Urochroa bougueri* | EU042594.1 | − | EU042509.1 | EU042429.1 |
| *Boissonneaua flavescens* | EU042530.1 | − | EU042448.1 | EU042366.1 |
| *Boissonneaua matthewsii* | AY830466.1 | − | AY830542.1 | AY830617.1 |
| *Aglaeactis cupripennis* | FJ903501.1 | − | AY830535.1 | AY830610.1 |
| *Aglaeactis castelnaudii* | AY830458.1 | − | AY830534.1 | AY830609.1 |
| *Aglaeactis pamela* | GU167201.1 | − | GU167147.1 | GU167091.1 |
| *Lafresnaya lafresnayi* | FJ903536.1 | − | AY830571.1 | AY830647.1 |
| *Coeligena coeligena* | FJ960684.1 | − | GU167163.1 | GU167107.1 |
| *Coeligena wilsoni* | FJ903532.1 | − | AY830551.1 | AY830626.1 |
| *Coeligena prunellei* | GU167221.1 | − | GU167167.1 | GU167111.1 |
| *Coeligena torquata* | AY830473.1 | − | − | − |
| *Coeligena phalerata* | FJ903521.1 | − | GU167166.1 | GU167110.1 |
| *Coeligena orina* | GU167219.1 | − | GU167165.1 | GU167109.1 |
| *Coeligena bonapartei* | GU167216.1 | KC858543.1 | GU167162.1 | GU167106.1 |
| *Coeligena helianthea* | FJ903511.1 | − | − | FJ903583.1 |
| *Coeligena lutetiae* | FJ903517.1 | − | EU042460.1 | EU042378.1 |
| *Coeligena violifer* | AY830474.1 | − | AY830550.1 | AY830625.1 |
| *Coeligena iris* | GU167218.1 | − | GU167164.1 | GU167108.1 |
| *Ensifera ensifera* | FJ903534.1 | − | AY830554.1 | AY830630.1 |
| *Pterophanes cyanopterus* | AY830520.1 | − | AY830595.1 | AY830671.1 |
| *Eriocnemis nigrivestis* | GU167227.1 | − | GU167173.1 | GU167117.1 |
| *Eriocnemis vestita* | EU042551.1 | − | GU166830.1 | EU042387.1 |
| *Eriocnemis cupreoventris* | GU167225.1 | − | GU167171.1 | GU167115.1 |
| *Eriocnemis luciani* | AY830480.1 | − | GU167171.1 | AY830631.1 |
| *Eriocnemis mosquera* | EU042550.1 | − | JQ267538.1 | EU042386.1 |
| *Eriocnemis glaucopoides* | EU042550.1 | − | GU167172.1 | GU167116.1 |
| *Eriocnemis alinae* | EU042549.1 | − | EU042467.1 | EU042385.1 |
| *Haplophaedia aureliae* | AY830487.1 | − | AY830562.1 | AY830638.1 |
| *Haplophaedia lugens* | EU042555.1 | − | EU042471.1 | EU042391.1 |
| *Urosticte ruficrissa* | GU167256.1 | − | GU167199.1 | GU167145.1 |
| *Ocreatus underwoodii* | AY830504.1 | − | AY830579.1 | AY830655.1 |
| *Heliomaster longirostris* | AY830493.1 | KC858652.1 | AY830568.1 | AY830644.1 |
| *Heliomaster constantii* | KC858529.1 | − | − | − |
| *Heliomaster furcifer* | GU167234.1 | − | GU167180.1 | GU167124.1 |

| *Coquettes* |  |  |  |  |
| --- | --- | --- | --- | --- |
| Specie | *ND2* | *ND5* | *AK1* | *Bfib* |

| *Lophornis delattrei* | AY830500.1 | − | AY830575.1 | AY830651.1 |
| --- | --- | --- | --- | --- |
| *Lophornis chalybeus* | GU167239.1 | − | GU167185.1 | GU167129.1 |
| *Lophornis pavoninus* | EU042568.1 | − | EU042483.1 | EU042403.1 |
| *Discosura popelairii* | EU042546.1 | − | EU042464.1 | EU042382.1 |
| *Discosura langsdorffi* | GU167223.1 | − | − | GU167113.1 |
| *Discosura conversii* | AY830519.1 | − | AY830594.1 | AY830670.1 |
| *Discosura longicaudus* | GU167224.1 | − | GU167170.1 | GU167114.1 |
| *Adelomyia melanogenys* | AY830457.1 | − | AY830533.1 | AY830608.1 |
| *Phlogophilus hemileucurus* | AY830517.1 | − | AY830592.1 | AY830668.1 |
| *Oreotrochilus chimborazo* | AY830506.1 | − | AY830581.1 | AY830657.1 |
| *Oreotrochilus estella* | AY830507.1 | − | AY830582.1 | − |
| *Oreotrochilus leucopleurus* | U85722.1 | − | − | − |
| *Oreotrochilus melanogaster* | GU166854.1 | − | GU166835.1 | GU166844.1 |
| *Sephanoides sephaniodes* | EU042592.1 | − | EU042507.1 | − |
| *Sephanoides fernandensis* | EU042591.1 | − | EU042506.1 | EU042426.1 |
| *Heliangelus amethysticollis* | AY830489.1 | − | AY830564.1 | AY830640.1 |
| *Heliangelus strophianus* | GU167231.1 | − | GU167177.1 | GU167121.1 |
| *Heliangelus exortis* | EU042556.1 | − | GU166831.1 | EU042392.1 |
| *Heliangelus micraster* | GU167230.1 | − | GU167176.1 | GU167120.1 |
| *Heliangelus viola* | GU167232.1 | − | GU167178.1 | GU167122.1 |
| *Heliangelus regalis* | GU166850.1 | − | GU166832.1 | GU166841.1 |
| *Lesbia victoriae* | AY830499.1 | − | AY830574.1 | AY830650.1 |
| *Lesbia nuna* | AY830498.1 | − | AY830573.1 | AY830649.1 |
| *Sappho sparganura* | GU167249.1 | − | GU167194.1 | GU167138.1 |
| *Polyonymus caroli* | GU166855.1 | − | GU166836.1 | GU166845.1 |
| *Ramphomicron microrhynchum* | EU042587.1 | − | EU042502.1 | EU042422.1 |
| *Oreonympha nobilis* | AY830505.1 | − | AY830580.1 | AY830656.1 |
| *Oxypogon guerinii* | EU042573.1 | − | − | − |
| *Metallura tyrianthina* | AY830502.1 | − | AY830577.1 | AY830653.1 |
| *Metallura williami* | EU042570.1 | − | EU042485.1 | EU042405.1 |
| *Metallura baroni* | GU167240.1 | − | GU167186.1 | GU167130.1 |
| *Metallura odomae* | GU167241.1 | − | GU167187.1 | GU167131.1 |
| *Metallura theresiae* | GU166853.1 | − | GU166834.1 | GU166843.1 |
| *Metallura eupogon* | GU166852.1 | − | GU166833.1 | GU166842.1 |
| *Metallura aeneocauda* | AY830501.1 | − | AY830576.1 | AY830652.1 |
| *Metallura phoebe* | EU042569.1 | − | EU042484.1 | EU042404.1 |
| *Chalcostigma ruficeps* | AY830469.1 | − | AY830545.1 | EU042404.1 |
| *Chalcostigma olivaceum* | GU166848.1 | − | GU166828.1 | GU166839.1 |
| *Chalcostigma stanleyi* | GU167255.1 | − | − | − |
| *Chalcostigma herrani* | EU042536.1 | − | EU042454.1 | EU042372.1 |
| *Opisthoprora euryptera* | EU042572.1 | − | EU042487.1 | EU042407.1 |
| *Taphrolesbia griseiventris* | GU166856.1 | − | GU166837.1 | GU166846.1 |
| *Aglaiocercus kingi* | AY830461.1 | − | GU166837.1 | AY830612.1 |
| *Aglaiocercus coelestis* | AY830460.1 | − | AY830536.1 | AY830611.1 |

| *Patagona* |  |  |  |  |
| --- | --- | --- | --- | --- |
| Specie | *ND2* | *ND5* | *AK1* | *Bfib* |

| *Patagona gigas* | AY830510.1 | AY830661.1 | AY830585.1 | − |
| --- | --- | --- | --- | --- |

| *Mtn. Gems* |  |  |  |  |
| --- | --- | --- | --- | --- |
| Specie | *ND2* | *ND5* | *AK1* | *Bfib* |

| *Lampornis viridipallens* | EU543355.1 | − | AY275885.1 | − |
| --- | --- | --- | --- | --- |
| *Lampornis sybillae* | EU543356.1 | DQ196559.1 | − | − |
| *Lampornis amethystinus* | EU543352.1 | − | KC858649.1 | − |
| *Lampornis clemenciae* | EU543354.1 | KC858648.1 | − | − |
| *Lampornis hemileucus* | EU042567.1 | DQ196586.1 | EU042482.1 | EU042402.1 |
| *Lampornis calolaemus* | EU042565.1 | DQ196563.1 | EU042480.1 | EU042400.1 |
| *Lampornis castaneoventris* | EU042566.1 | DQ196580.1 | EU042481.1 | EU042401.1 |
| *Lamprolaima rhami* | EU543358.1 | KC858650.1 | − | − |
| *Eugenes fulgens* | AY830481.1 | KC858651.1 | AY830556.1 | AY830632.1 |
| *Panterpe insignis* | AY830509.1 | − | AY830584.1 | AY830660.1 |

| *Bees* |  |  |  |  |
| --- | --- | --- | --- | --- |
| Specie | *ND2* | *ND5* | *AK1* | *Bfib* |

| *Rhodopis vesper* | EU042588.1 | − | EU042503.1 | EU042423.1 |
| --- | --- | --- | --- | --- |
| *Thaumastura cora* | JQ025424.1 | − | JQ025410.1 | GU167141.1 |
| *Calliphlox bryantae* | EU042531.1 | − | EU042449.1 | EU042367.1 |
| *Calliphlox amethystina* | GU167211.1 | − | GU167157.1 | GU167101.1 |
| *Microstilbon burmeisteri* | GU167242.1 | − | GU167188.1 | GU167132.1 |
| *Doricha eliza* | KC858530.1 | KC858653.1 | − | − |
| *Tilmatura dupontii* | KC858535.1 | KC858657.1 | − | − |
| *Calothorax lucifer* | KC858532.1 | KC858654.1 | − | − |
| *Calothorax pulcher* | KC858531.1 | AY275889.1 | − | − |
| *Myrtis fanny* | AY830503.1 | − | AY830578.1 | AY830654.1 |
| *Eulidia yarrellii* | JQ025412.1 | − | JQ025416.1 | JQ025415.1 |
| *Myrmia micrura* | GU167243.1 | − | GU167189.1 | GU167133.1 |
| *Archilochus colubris* | AY830465.1 | KC858655.1 | AY830541.1 | AY830616.1 |
| *Archilochus alexandri* | EU042529.1 | − | EU042447.1 | EU042365.1 |
| *Calypte anna* | EU042532.1 | − | EU042450.1 | EU042368.1 |
| *Calypte costae* | EU042533.1 | − | EU042451.1 | EU042369.1 |
| *Stellula calliope* | KF792866.1 | − | − | − |
| *Atthis heloisa* | KC858534.1 | AY275887.1 | − | − |
| *Chaetocercus mulsant* | AY830456.1 | − | AY830532.1 | AY830607.1 |
| *Chaetocercus bombus* | GU167213.1 | − | GU167159.1 | GU167103.1 |
| *Selasphorus platycercus* | AY830522.1 | − | AY830597.1 | AY830673.1 |
| *Selasphorus rufus* | EU042590.1 | KC858656.1 | EU042505.1 | EU042425.1 |
| *Selasphorus sasin* | KF792865.1 | − | − | − |
| *Selasphorus flammula* | EU042589.1 | − | EU042504.1 | EU042424.1 |

| *Emeralds* |  |  |  |  |
| --- | --- | --- | --- | --- |
| Specie | *ND2* | *ND5* | *AK1* | *Bfib* |

| *Phaeochroa cuvierii* | GU167244.1 | − | GU167190.1 | GU167134.1 |
| --- | --- | --- | --- | --- |
| *Campylopterus largipennis* | KC858431.1 | KC858553.1 | AY830543.1 | AY830618.1 |
| *Campylopterus rufus* | KC858429.1 | − | − | − |
| *Campylopterus hemileucurus* | KC858430.1 | KC858552.1 | EU042452.1 | EU042370.1 |
| *Campylopterus hyperythrus* | EU042535.1 | − | − | EU042371.1 |
| *Campylopterus curvipennis* | KC858426.1 | − | − | − |
| *Campylopterus falcatus* | GU167212.1 | − | − | GU167102.1 |
| *Campylopterus villaviscensio* | AY830468.1 | KC858554.1 | AY830544.1 | AY830619.1 |
| *Eupetomena macroura* | AY115471.1 | − | GU167174.1 | GU167118.1 |
| *Aphantochroa cirrochloris* | EU042528.1 | − | EU042446.1 | EU042364.1 |
| *Orthorhyncus cristatus* | AY830508.1 | − | AY830583.1 | AY830659.1 |
| *Klais guimeti* | AY830495.1 | − | AY830570.1 | AY830646.1 |
| *Stephanoxis lalandi* | GU167250.1 | − | GU167195.1 | GU167139.1 |
| *Chlorostilbon melanorhynchus* | AY830470.1 | − | AY830546.1 | AY830621.1 |
| *Chlorostilbon mellisugus* | AY830471.1 | − | AY830547.1 | AY830622.1 |
| *Chlorostilbon canivetii* | KC858433.1 | KC858555.1 | − | − |
| *Chlorostilbon aureoventris* | GU167214.1 | − | GU167160.1 | GU167104.1 |
| *Chlorostilbon poortmani* | GU167215.1 | − | GU167161.1 | GU167105.1 |
| *Cynanthus sordidus* | EU418755.1 | KC858556.1 | − | − |
| *Cynanthus latirostris* | EU418745.1 | KC858557.1 | − | − |
| *Cyanophaia bicolor* | EU418756.1 | − | − | − |
| *Thalurania colombica* | AY830524.1 | − | AY830599.1 | AY830675.1 |
| *Thalurania fannyi* | GU167257.1 | − | − | GU167146.1 |
| *Thalurania furcata* | JQ445835.1 | − | AY830600.1 | AY830676.1 |
| *Thalurania glaucopis* | GU167251.1 | − | GU167196.1 | GU167140.1 |
| *Juliamyia julie* | EU042545.1 | KC858545.1 | EU042463.1 | EU042381.1 |
| *Lepidopyga coeruleogularis* | AY830497.1 | KC858546.1 | AY830572.1 | AY830648.1 |
| *Lepidopyga goudoti* | JQ267532.1 | − | JQ267539.1 | − |
| *Hylocharis eliciae* | EU042562.1 | KC858558.1 | EU042478.1 | EU042398.1 |
| *Hylocharis sapphirina* | EU042564.1 | KC858559.1 | EU042479.1 | EU042399.1 |
| *Hylocharis cyanus* | EU042561.1 | KC858561.1 | EU042477.1 | EU042397.1 |
| *Hylocharis chrysura* | GU167236.1 | KC858560.1 | GU167182.1 | GU167126.1 |
| *Hylocharis grayi* | EU042563.1 | − | − | − |
| *Hylocharis leucotis* | EU418759.1 | KC858562.1 | − | − |
| *Hylocharis xantusii* | EU543353.1 | DQ196590.1 | − | − |
| *Chrysuronia oenone* | AY830472.1 | KC858547.1 | AY830548.1 | AY830623.1 |
| *Polytmus guainumbi* | EU042585.1 | − | EU042500.1 | EU042420.1 |
| *Polytmus theresiae* | EU042586.1 | − | EU042501.1 | EU042421.1 |
| *Leucippus baeri* | GU167237.1 | KC858564.1 | GU167183.1 | GU167127.1 |
| *Leucippus taczanowskii* | KC858441.1 | KC858563.1 | − | − |
| *Leucippus chlorocercus* | GU167238.1 | − | GU167184.1 | GU167128.1 |
| *Taphrospilus hypostictus* | AY830523.1 | − | AY830598.1 | AY830674.1 |
| *Amazilia chionogaster* | AY830462.1 | KC858575.1 | AY830538.1 | AY830613.1 |
| *Amazilia viridicauda* | GU167206.1 | KC858576.1 | GU167152.1 | GU167096.1 |
| *Amazilia amazilia* | GU167202.1 | KC858566.1 | GU167148.1 | GU167092.1 |
| *Amazilia candida* | KC858461.1 | KC858585.1 | − | − |
| *Amazilia brevirostris* | KC858446.1 | KC858567.1 | − | − |
| *Amazilia versicolor* | EU042525.1 | KC858586.1 | EU042443.1 | − |
| *Amazilia franciae* | EU042521.1 | KC858573.1 | EU042439.1 | EU042357.1 |
| *Amazilia fimbriata* | EU042520.1 | KC858587.1 | EU042438.1 | EU042356.1 |
| *Amazilia lactea* | GU167203.1 | KC858588.1 | GU167149.1 | GU167093.1 |
| *Amazilia amabilis* | EU042518.1 | KC858589.1 | EU042436.1 | EU042354.1 |
| *Amazilia decora* | EU042519.1 | KC858569.1 | EU042437.1 | EU042355.1 |
| *Amazilia rosenbergi* | GU167204.1 | − | GU167150.1 | GU167094.1 |
| *Amazilia cyanocephala* | KC858468.1 | KF640767.1 | − | − |
| *Amazilia beryllina* | EU418761.1 | KC858613.1 | − | − |
| *Amazilia cyanura* | KC858491.1 | KF640750.1 | − | − |
| *Amazilia saucerrottei* | EU042523.1 | KC858615.1 | GU167151.1 | GU167095.1 |
| *Amazilia edward* | KC858494.1 | KC858617.1 | − | − |
| *Amazilia viridigaster* | EU042526.1 | KC858616.1 | GU167153.1 | EU042362.1 |
| *Amazilia tzacatl* | EU983387.1 | KC858635.1 | EU042442.1 | EU042360.1 |
| *Amazilia yucatanensis* | KC858506.1 | KC858629.1 | − | EU042360.1 |
| *Amazilia rutila* | EU042522.1 | KC858622.1 | EU042440.1 | EU042358.1 |
| *Amazilia violiceps* | KC858514.1 | KC858638.1 | − | − |
| *Amazilia viridifrons* | KC858521.1 | KC858644.1 | KC858638.1 | − |
| *Amazilia tobaci* | KC858448.1 | KC858570.1 | − | AY695143.1 |
| *Eupherusa eximia* | EU042552.1 | KC858646.1 | EU042468.1 | EU042388.1 |
| *Eupherusa cyanophrys* | KC858523.1 | KC858647.1 | − | − |
| *Eupherusa poliocerca* | KC858522.1 | KC858645.1 | − | − |
| *Eupherusa nigriventris* | EU042553.1 | − | EU042469.1 | EU042389.1 |
| *Elvira chionura* | EU042548.1 | − | EU042466.1 | EU042384.1 |
| *Elvira cupreiceps* | AY830478.1 | − | AY830553.1 | AY830629.1 |
| *Microchera albocoronata* | EU042571.1 | − | EU042486.1 | EU042406.1 |
| *Anthocephala floriceps* | GU167208.1 | − | GU167154.1 | GU167098.1 |
| *Chalybura buffonii* | EU042537.1 | − | EU042455.1 | EU042373.1 |
| *Chalybura urochrysia* | EU042538.1 | − | EU042456.1 | EU042374.1 |
